# Isolated metachronous splenic metastasis after rectal cancer surgery: a case report and literature review

**DOI:** 10.3389/fonc.2025.1563632

**Published:** 2025-08-07

**Authors:** Peide Ren, Yihang Shi, Letian Zhang, Yuemin Sun, Zhibin Ye, Hengchang Liu, Shunda Wang, Yinggang Chen

**Affiliations:** ^1^ Department of Gastrointestinal Surgery, Cancer Hospital Shenzhen Hospital, Chinese Academy of Medical Sciences and Peking Union Medical College, Shenzhen, China; ^2^ Department of Colorectal Surgery, National Cancer Center/National Clinical Research Center for Cancer/Cancer Hospital, Chinese Academy of Medical Sciences and Peking Union Medical College, Beijing, China; ^3^ Department of Gastrointestinal surgery, Hebei General Hospital, Shijiazhuang, Hebei, China

**Keywords:** colorectal cancer, isolated splenic metastasis, metachronous, laparoscopic splenectomy, literature review

## Abstract

**Background:**

The occurrence of isolated splenic metastasis in association with colorectal cancer is exceedingly rare. This paper presents a case of isolated splenic metastasis identified one year after radical resection of rectal cancer. The patient underwent laparoscopic splenectomy, achieving favorable therapeutic outcomes. Furthermore, we conduct a literature review of analogous cases and examine the clinical diagnosis and therapeutic approaches for this uncommon condition.

**Case Review:**

A 76-year-old male patient underwent laparoscopic radical resection for rectal cancer, accompanied by ileostomy, at our institution. Postoperatively, the patient received two cycles of XELOX chemotherapy but continued with oral capecitabine for six cycles due to oxaliplatin intolerance. Follow-up enhanced CT scans of the chest, abdomen, and pelvis revealed an isolated metastatic tumor in the spleen. Subsequently, the patient underwent laparoscopic splenectomy. Histopathological examination post-surgery confirmed the metastasis of rectal cancer to the spleen.

**Conclusion:**

Isolated splenic metastasis originating from colorectal cancer is exceedingly rare, and laparoscopic splenectomy constitutes an effective treatment strategy.

## Background

Colorectal cancer is currently the third most common malignancy worldwide. Despite its prevalence, splenic metastasis from colorectal cancer is rare.

Splenic metastasis is an exceedingly rare condition, largely attributable to the spleen’s unique anatomical structure and immunological features (1, 2). Anatomically, the splenic artery branches at an acute angle from the celiac artery, the splenic sinuses undergo rhythmic contraction, and the spleen lacks afferent lymphatic vessels. Immunologically, the spleen’s microenvironment, abundant in macrophages and monocytes, further impedes the development of metastatic tumors.

Based on the timing of splenic metastasis relative to the primary tumor, splenic metastases are classified as either synchronous or metachronous. Synchronous splenic metastases are defined in the literature as those identified concurrently with the primary tumor on imaging studies. Metachronous splenic metastases, in contrast, are those primarily detected during postoperative follow-up of the primary tumor lesion (3). However,the classification of splenic metastases found shortly after primary tumor resection—whether as synchronous or metachronous—remains controversial and lacks a definitive consensus. Drawing a parallel from the definition applied to liver metastases, metastases detected within 6 months following primary tumor resection are still classified as synchronous in the hepatic context. Critically, synchronous and metachronous hepatic metastases exhibit distinctly different patterns of incidence over time and divergence in survival outcomes. Therefore, a precise definition of synchronous splenic metastasis will likely necessitate future studies analyzing the clinical outcomes and long-term prognosis of a larger cohort of patients developing splenic metastases after resection of the primary tumor (4). In this patient, the splenic metastasis was detected at 1 year following resection of the primary tumor, consistent with a metachronous presentation.

When splenic metastasis does occur, it is typically associated with metastasis to other sites, rendering the incidence of isolated splenic metastasis even more uncommon. At present, there is a paucity of large-scale randomized controlled trials providing evidence for the treatment of splenic metastases, with current knowledge primarily derived from case reports.

This article presents a rare case of isolated splenic metastasis and reviews pertinent literature on isolated splenic metastasis associated with colorectal cancer, sourced from the PubMed database. The objective is to elucidate the mechanisms underlying the occurrence, diagnosis, and treatment of splenic metastasis, thereby offering additional references for future clinical management.

## Case description

### Splenic metastasis detected following curative resection of rectal cancer

**Figure 1:**
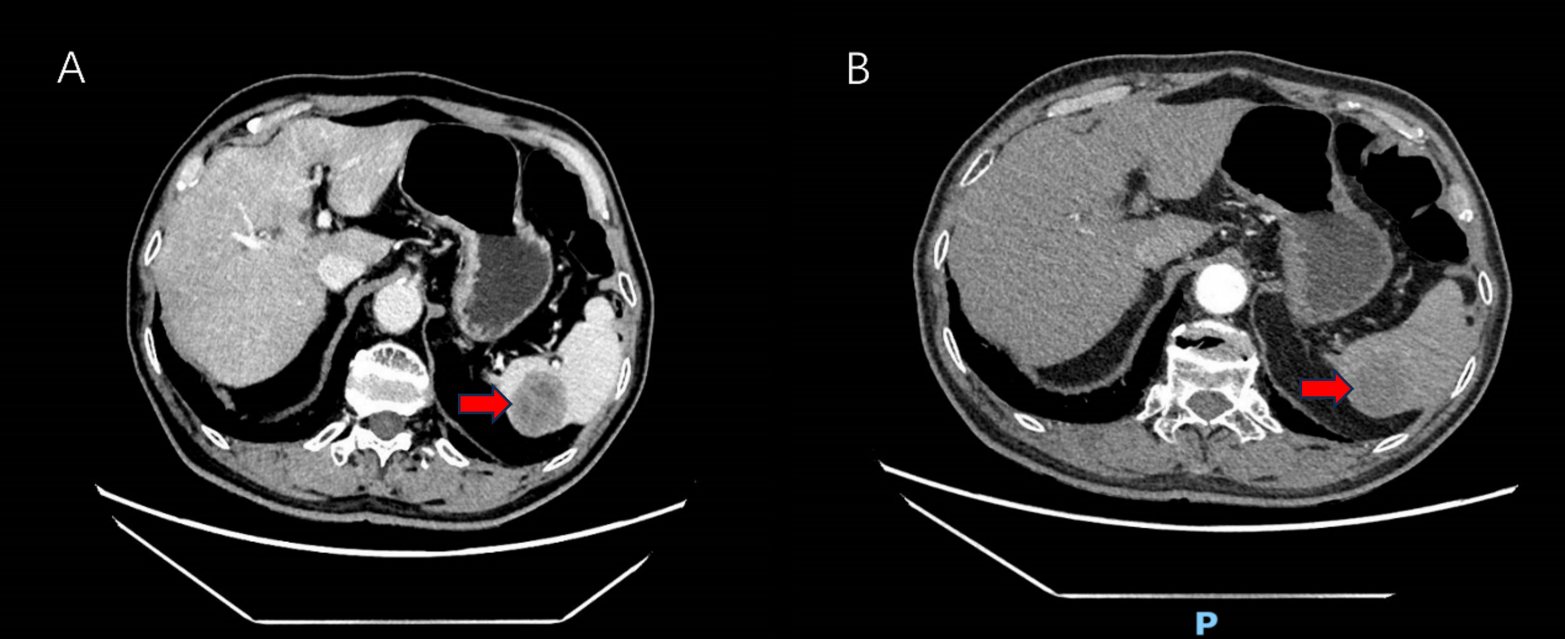
Abdomen enhanced CT showing low-density shadows in the spleen (red arrow). **(A)** Venous-phase splenic metastasis shows a hypodense lesion (red arrow). **(B)** Arterial-phase CT reveals a hypodense lesion with peripheral enhancement (red arrow).

A 76-year-old male patient, with a medical history significant for hypertension, atrial fibrillation, and cerebral infarction and no familial predisposition to tumors, underwent laparoscopic radical resection for rectal cancer and a prophylactic ileostomy on January 6, 2023. Postoperative pathological analysis revealed a classification of pT4aN1b, accompanied by tumor budding and poorly differentiated tumor cell clusters, with evidence of vascular and neural invasion. Immunohistochemical analysis demonstrated the following results: BRAF-V600E negative, C-MET 1+, HER2 1+, MLH1 positive, MSH2 positive, MSH6 positive, and PMS2 positive, with a microsatellite status of stable (MSS). The patient was administered two cycles of adjuvant chemotherapy according to the XELOX regimen; however, due to oxaliplatin intolerance, treatment was continued with oral capecitabine for an additional six cycles. On September 19, 2023, the patient underwent ileostomy reversal. Routine follow-up assessments, including carcinoembryonic antigen (CEA), carbohydrate antigen 19-9 (CA19-9), carbohydrate antigen 242 (CA242), and enhanced computed tomography (CT) scans of the chest, abdomen, and pelvis, indicated normal parameters with no signs of tumor recurrence. However, on August 8, 2024, laboratory tests revealed elevated levels of CEA, CA19-9, and CA242. Subsequent CT imaging on August 14, 2024, identified a new lesion in the spleen, measuring approximately 4.0 × 3.7 cm (**Figure 1**). Mildly irregular, suggesting a metastatic tumor. However, there was no evidence of metastasis to the liver, lungs, or peritoneum. Given the potential for isolated metachronous splenic metastasis and the patient’s intolerance to oxaliplatin, a multidisciplinary team (MDT) discussion resulted in the decision to perform a laparoscopic splenectomy under general anesthesia on August 26, 2024.

### Surgical treatment

**Figure 2:**
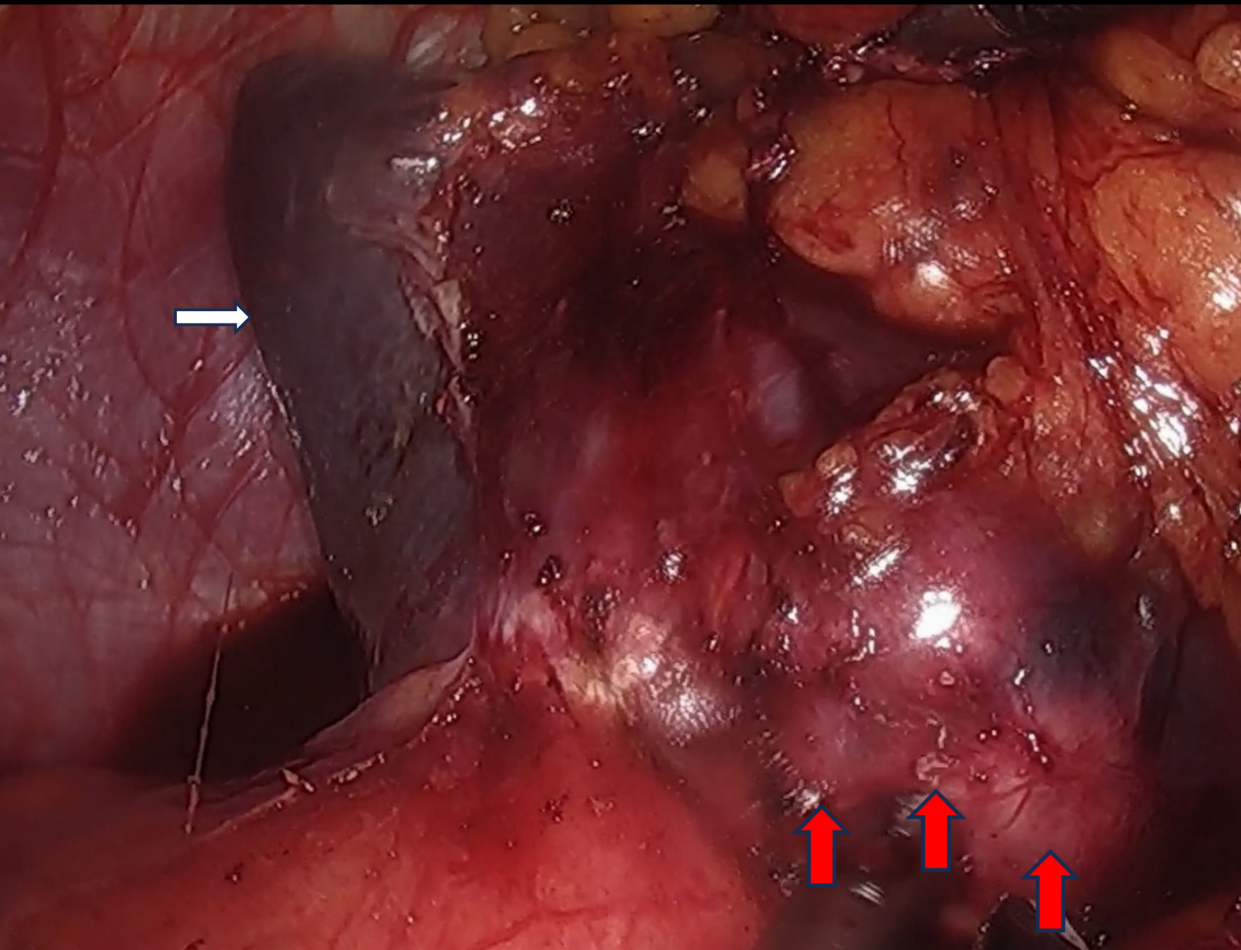
Laparoscopic exploration of a hard tumor in the spleen (The white arrow indicates the spleen, the red arrow denotes the metastatic lesion within the spleen).

After consent was obtained from the patient and his family, the procedure was performed. We conducted a five-port laparoscopic procedure.Intraoperative exploration revealed the absence of metastatic nodules in the liver, peritoneum, or pelvis, and no ascites was present. However, a firm, white nodule was observed at the lower pole of the spleen (**Figure 2**). So we performed a laparoscopic total splenectomy and placed a drainage tube in the splenic fossa.The surgery went smoothly without any intraoperative complications.

### Postoperative pathology

**Figure 3:**
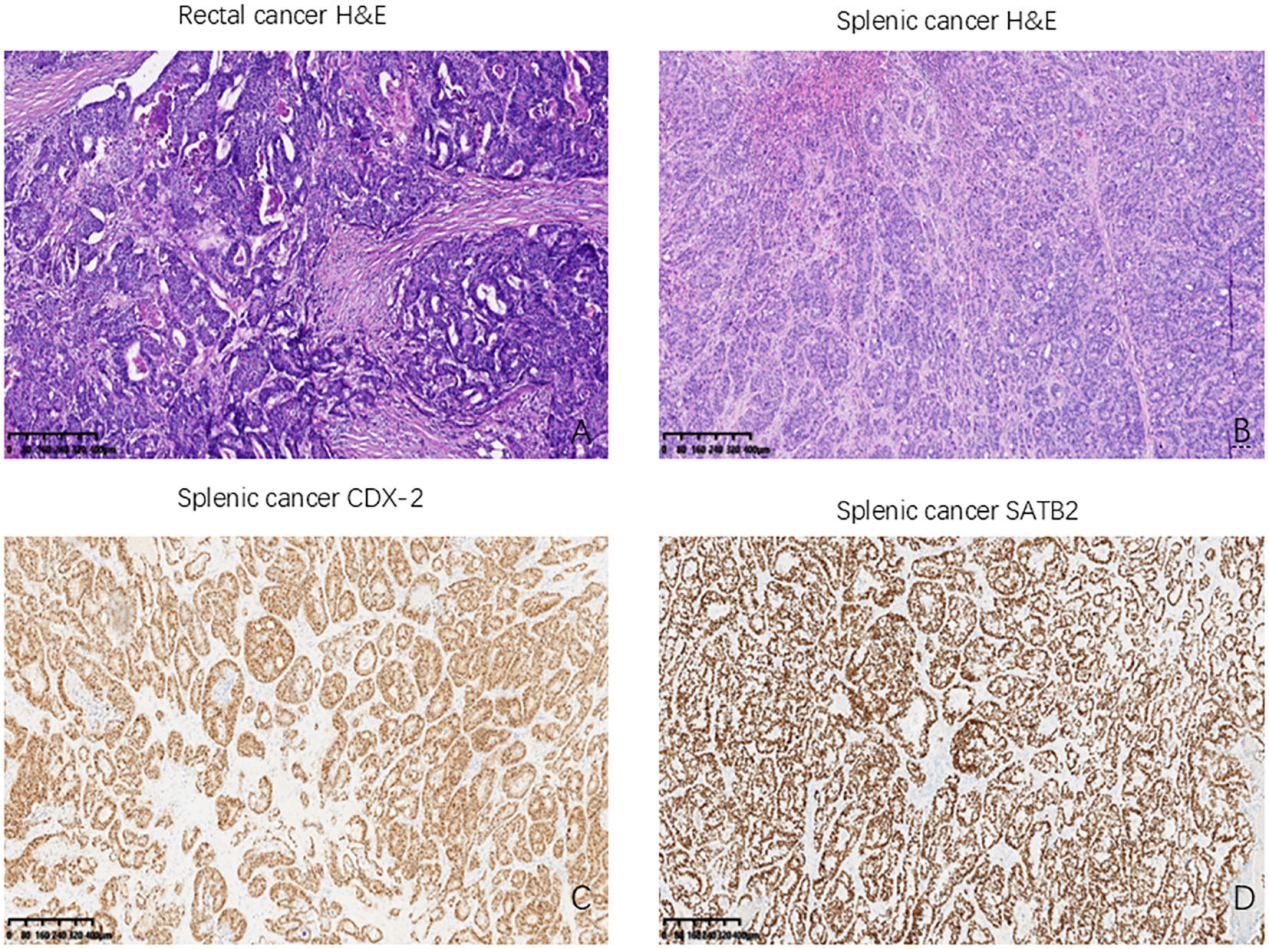
Histological results of the patient we reported. **(A)** The primary rectal cancer: Poorly differentiated adenocarcinoma (HE, ×400); **(B)** Splenic metastasis from rectal cancer (HE, ×400); **(C)** CDX-2 positively stained in splenic metastasis (CDX2, ×400); **(D)** SATB2 positively stained in splenic metastasis (SATB2, ×400). HE: hematoxylin and eosin, CDX-2: Homeobox Transcription Factor 2; SATB2: Special AT-rich sequence-binding protein 2.

The postoperative pathological findings from the previous radical resection for rectal cancer demonstrated poorly differentiated adenocarcinoma (**Figure 3A**). Postoperative pathological analysis identified the nodule as moderately to poorly differentiated intestinal-type adenocarcinoma infiltrating the splenic parenchyma, with extensive necrosis and focal mucin secretion (**Figure 3B**). Correlating these findings with the patient’s medical history, morphological characteristics, and immunohistochemical results, the diagnosis was consistent with splenic metastasis originating from colorectal adenocarcinoma. The tumor involved the splenic capsule but did not extend into the splenic hilum or omentum. No definitive vascular or neural invasion was detected. The surrounding splenic tissue appeared unremarkable. The pathological staging was pTNM: pM1, to be interpreted in conjunction with clinical data. Immunohistochemical analysis demonstrated the following results: CDX-2 (2+), CD10 (3+), SATB2 (3+), Villin (3+), CK7 (-), and CK20 (-) (refer to **Figures 3C, D**). Postoperatively, the patient exhibited no fever and no elevation in platelet count (PLT). And the patient was discharged one week after the procedure. It was advised that the patient commence oral capecitabine therapy three weeks following surgery. At present, the patient is maintaining a favorable follow-up status.

## Discussion

Colorectal cancer ranks as the third most prevalent cancer globally and is associated with the fourth highest mortality rate (5). Metastasis accounts for approximately 90% of colorectal cancer-related deaths (6). The liver is the most frequent site of distant metastasis, followed by lymph nodes, lungs, and peritoneum, while metastases to the brain and bones are uncommon (7). The incidence of splenic metastasis is relatively low. A comprehensive autopsy study conducted by Professor Berge reported a 7.1% incidence of splenic metastases among cancer patients, with colorectal cancer exhibiting a 4.4% likelihood of splenic metastasis, which is lower than that observed in skin, breast, and ovarian cancers. However, the study did not address the incidence of isolated splenic metastasis (6). Professor Berge’s research identified that among cases exhibiting metastasis in five or more organs, 50% presented with microscopic splenic metastases. In comparison to subjects lacking splenic metastasis, the incidence of lymph node and other organ metastases was significantly lower (8). Consequently, splenic metastasis is often indicative of advanced disease. However, unlike splenic metastases associated with widespread metastases throughout the body, solitary splenic metastases do not signify the terminal stages of metastatic cancer. Instead, they reflect the proliferation of cancer cells in the spleen during the latent phase of early hematogenous dissemination (9). Indeed, the likelihood of isolated splenic metastasis is exceedingly low. Another study reported that among 29,364 cancer patients, 20.9% had metastases, yet only 59 patients exhibited splenic metastases (0.2% of the total cohort), and merely three patients had isolated splenic metastases (10). As previously mentioned, there is currently no definitive explanation for the relatively low incidence of splenic metastasis, although several hypotheses have been proposed in the academic community (1, 8, 11–16). First, the sharp angulation of the splenic artery at its origin from the celiac trunk impedes tumor emboli migration toward the spleen (8, 15). Second, rhythmic splenic contractions prevent colonization of tumor emboli (17). Third, the absence of afferent lymphatic vessels reduces tumor trafficking to the spleen (15). Finally, the immunologically active splenic microenvironment—abundant in macrophages and monocytes—inhibits metastatic development (16).

**Figure 4:**
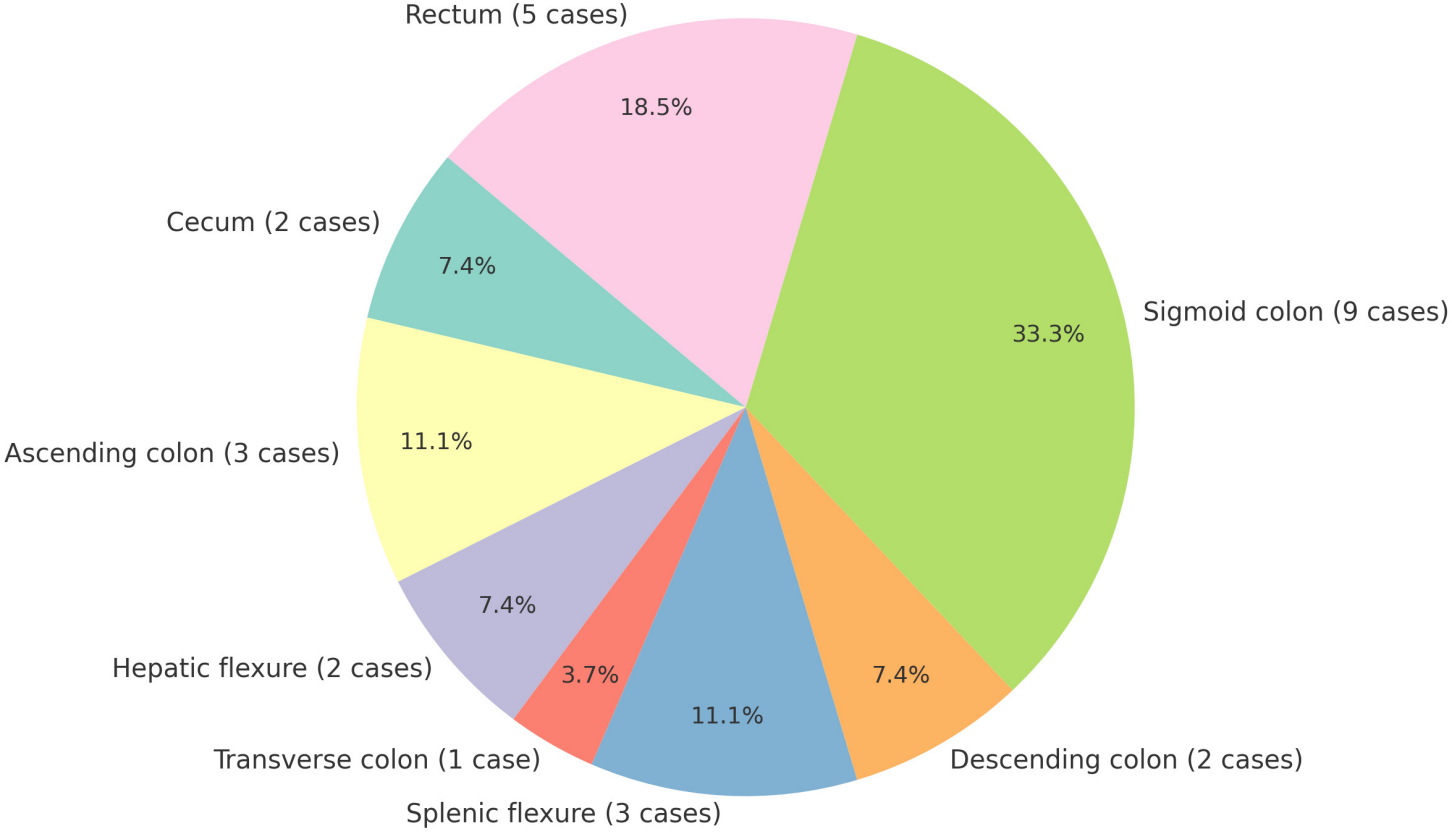
Distribution of primary tumor sites in metastatic spleen cases [Sigmoid colon was the most frequent site of involvement (33.3%, 9/27)].

Through systematic literature searches conducted in PubMed, Embase, Web of Science, and the Cochrane Library using the keywords”colon cancer,” “rectal cancer,” “colorectal cancer,” and “splenic metastasis,” we identified only 27 documented case reports of isolated splenic metastases secondary to colorectal carcinoma (**Table 1**). The earliest documented case was reported by Professor Dunbar in 1969 (18). Among the cases examined, 6 involved synchronous splenic metastasis, while 21 involved metachronous splenic metastasis. The statement indicates that metachronous splenic metastasis is more prevalent than synchronous splenic metastasis. The cohort comprised 15 female and 12 male patients, with ages ranging from 33 to 84 years. The primary tumor sites included the cecum (2 case), ascending colon (3 cases), hepatic flexure (2 cases), transverse colon (1 case), splenic flexure (3 cases), descending colon (2 case), sigmoid colon (9 cases), and rectum (5 cases) (**Figure 4**). The sigmoid colon was the most frequent site of involvement (33.3%), followed by the rectum (18.5%). Splenic metastasis demonstrates a pronounced predilection for malignancies arising from the left colon and rectum, a clinicopathologic pattern attributable to vascular anatomic determinants. Tumor cells undergo retrograde venous transmission via the inferior mesenteric vein, directly accounting for both the predominantly intraparenchymal localization of metastatic deposits and their increased frequency in left-sided versus right-sided carcinomas (12). Notably, the splenic parenchyma lacks afferent lymphatics, structurally reinforcing hematogenous dissemination as the principal pathway (19). Contemporary consensus affirms this mechanistic dominance despite coexisting lymphatic routes (8, 19, 20).

**Table 1:** Review of the isolate splenic metastases from CRC cases.

No.	Year	Age (years)	Gender	Primary tumor	Stage	CEA level (ng/ml)	Interval* (months)	Diagnosis methods for splenic metastasis	Treatment for Splenic metastasis	DFS (months)	Ref
1	1969	78	F	Rectum	III	NA	48	CT	Surgery	84	(18)
2	1992	51	F	Rectum	II	13.5	51	CT	Surgery	14	(37)
3	1997	74	M	Sigmoid	NA	23.4	24	CT	Surgery	24	(38)
4	1999	41	M	Rectum	NA	NA	12	Open exploratory surgery	Surgery	NA	(19)
5	1999	33	F	Sigmoid	III	34.1	3	CT MRI	Surgery	12	(39)
6	2001	62	M	Sigmoid	III	2.5	23	CT	Surgery	19	(11)
7	2001	51	M	Sigmoid	III	5.0	72	CT and needle biopsy	Surgery	NA	(20)
8	2003	73	F	Descending colon	II	NA	72	CT	Surgery	NA	(40)
9	2006	76	M	Sigmoid	III	35	14	PET/CT	Surgery	12	(14)
10	2007	54	F	Splenic flexure	IV	31.1	Syn	CT	Surgery, CMT	6	(12)
11	2007	69	F	Sigmoid	II	20	24	CT	Surgery	60	(41)
12	2008	80	F	Transverse	III	52.3	9	CT	Surgery	no long-term follow-up	(1)
13	2009	73	M	Hepatic flexure	III	53.2	60	CT PET/CT	Surgery	40	(25)
14	2010	70	M	Splenic flexure	III	5.05	24	CT and biopsy	Surgery	10	(21)
15	2010	59	M	Ascending colon	III	37	15	CT	Surgery	24	(42)
16	2011	46	M	Ascending colon	III	152	NA	CT	Surgery	36	(26)
17	2012	74	M	Splenic flexure	IV	242.47	Syn	CT	Surgery, CMT	no long-term follow-up	(15)
18	2016	64	F	Cecum	I	38	16	CT	Surgery	6	(43)
19	2016	62	F	Sigmoid	III	NA	36	CT	Surgery	10	(17)
20	2017	76	F	Descending colon	III	Normal	28	PET-CT	Surgery	21	(44)
21	2019	73	M	Hepatic flexure	IV	6.9	Syn	CT	Surgery	6	(13)
22	2020	48	F	Sigmoid	III	206.8	21	PET-CT/MR	Surgery, CMT	7	(45)
23	2021	83	F	Cecum	IV	NA	Syn	CT	Surgery	24	(46)
24	2022	82	F	Sigmoid	IV	NA	Syn	PET	Surgery	4	(47)
25	2022	84	F	Rectum	IV	57.57	Syn	CT	Surgery, CMT	no long-term follow-up	(3)
26	2023	41	F	Ascending colon	NA	5.5	24	CT and biopsy	CMT, Surgery	no long-term follow-up	(22)
27	2023	41	M	Rectum	III	NA	55	CT	Surgery	3	(2)
28	2024	76	M	Rectum	III	82.30	19	CT	Surgery, CMT	no long-term follow-up	Our case

NA, not available; CMT, chemotherapy; DFS, disease- free survival after splenectomy; CRC, colorectal cancer; F, female; M, male.

Interval*, The time from colorectal cancer surgery to the first detection of splenic metastasis.

Our case exemplifies this paradigm: A histologically confirmed solitary intraparenchymal splenic metastasis developed 19 months post-resection of an upper rectal adenocarcinoma, without hilar nodal involvement,Critical pathologic correlates(including absence of vascular tumor emboli and perineural invasion)further corroborate hematogenous spread. This spatiotemporal profile (primary tumor venous drainage via inferior mesenteric vasculature + latency interval) aligns precisely with established hemodynamic models of splenic seeding.

**Figure 5:**
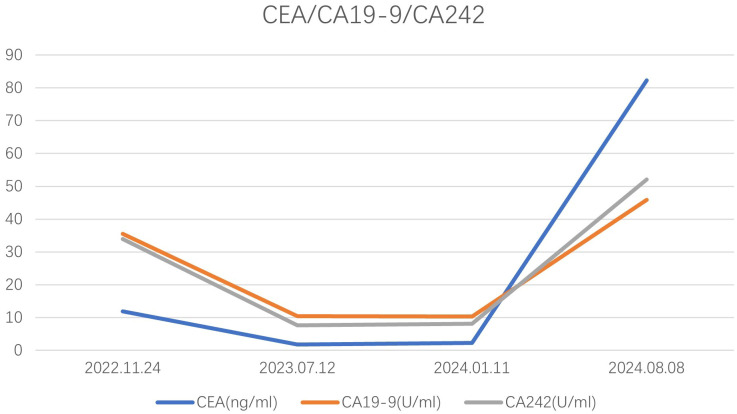
The concentration levels of CEA, CA19-9, and CA242 for the patient we reported at different time.

Notably, most patients with splenic metastasis are asymptomatic; however, one case was identified due to abdominal pain resulting from splenic rupture (19). The primary method of detection involves regular postoperative follow-up, which may reveal elevated carcinoembryonic antigen (CEA) levels or abnormalities on abdominal computed tomography (CT) scans. Among the reported patients, 80% exhibited elevated CEA levels, with the exception of five cases where CEA levels were not specified. In our case, the patient’s CEA level was 82.30 ng/ml, and elevated levels of CA19–9 and CA242 were also observed (**Figure 5**). Considering the initial preoperative elevation of CEA, its normalization following surgery, and subsequent elevation during splenic metastasis, we propose that dynamic monitoring of CEA levels can serve as a valuable tool for recurrence surveillance. Additionally, we identified several diagnostic modalities for detecting splenic metastasis, including abdominal ultrasound, fine-needle aspiration biopsy (20, 21), abdominal CT, abdominal magnetic resonance imaging (MRI), and positron emission tomography/computed tomography (PET/CT). Percutaneous biopsy enables pathological diagnosis that clarifies the nature of splenic lesions and informs subsequent therapeutic planning, albeit with inherent risks of hemorrhage and splenic abscess formation (20–22).PET/CT, due to its ability to rule out distant metastasis, plays a significant role in surgical decision-making, thereby increasing its utility in the diagnosis of splenic metastasis.

For metastatic colorectal cancer, treatment strategies encompass surgery, chemotherapy, targeted therapy, and radiotherapy. In the context of synchronous metastatic colorectal cancer, current consensus suggests that achieving an R0 resection should aim for a comprehensive tumor-free state, addressing both primary and metastatic lesions to enhance survival outcomes (23). For metachronous metastatic colorectal cancer, if the metastatic lesions are amenable to resection, the primary strategy involves excising these metastases initially. In cases where the lesions are not resectable, systemic therapy should be prioritized. Patients with initially unresectable disease should undergo follow-up evaluations every two months to reassess resectability. If resectability is achieved, surgical intervention is advised, followed by either observation or systemic therapy (3, 23, 24). Presently, there is limited evidence-based data regarding splenic metastases. However, for solitary liver or lung metastases from colorectal cancer, surgical resection has been shown to extend survival (23). As previously noted, solitary splenic metastasis does not necessarily indicate terminal metastatic disease. Case reports have suggested that surgical resection of solitary splenic metastasis can significantly prolong disease-free survival (25, 26). Furthermore, untreated splenic metastases may result in splenic rupture (19, 27). In the context of colorectal cancer splenic metastases, the adoption of laparoscopic surgery remains a subject of debate, primarily due to concerns regarding the potential risk of peritoneal dissemination. Nonetheless, certain studies suggest that laparoscopic surgery for colorectal cancer does not elevate the risk of intra-abdominal dissemination of cancer cells compared to conventional surgery (28). Moreover, laparoscopic splenectomy, in contrast to open surgery, is associated with reduced trauma, shorter hospitalization, expedited recovery, and lower rates of postoperative complications (29, 30). Laparoscopy also provides an opportunity to assess for peritoneal metastases. In our case, the patient presented with isolated splenic metastasis following laparoscopic radical resection of rectal cancer. Following a multidisciplinary team (MDT) discussion and consideration of the patient’s preferences, a laparoscopic splenectomy was performed. Intraoperatively, no hepatic or peritoneal metastases were detected, and the metastatic tumor was localized at the lower pole of the spleen. We removed the splenic metastatic tumor through the ileostomy closure incision to minimize wound size and recommended the patient continue oral capecitabine treatment. The patient is currently in good condition. Surgical treatment for isolated splenic metastases has shown promising results, with some patients achieving up to 7 years of disease-free survival (18).

For splenic metastases, treatment options extend beyond surgery and may include systemic chemotherapy, targeted therapy, immunotherapy, and ablation therapies. Currently, the evidence base supporting systemic treatment specifically for splenic metastases is limited, largely confined to case reports (31–34). The principles guiding systemic therapy for splenic metastases align with the overall treatment strategy for metastatic gastrointestinal (GI) cancers, with the primary objective being control of systemic disease.Available case reports also suggest the feasibility of medical therapy, indicating that chemotherapy can be safely administered in patients with pancytopenia secondary to hypersplenism, provided there is vigilant monitoring and supportive care (32).However, the emergence of anemia and thrombocytopenia during immunotherapy necessitates careful differentiation to determine their etiology: whether they are disease-related (due to metastases), treatment-related (attributable to chemotherapy/targeted agents), or immune-related toxicities.For severe bone marrow suppression or refractory hypersplenism, multidisciplinary team (MDT) consultation is strongly recommended to evaluate the need for splenectomy or interventional ablation.Ablation techniques for splenic metastases can serve as a curative treatment modality. The literature reports that radiofrequency ablation (RFA) and microwave ablation (MWA) display a safety profile that preserves splenic function while significantly reducing the risk of post-splenectomy infections and other associated complications (33). Nevertheless, splenectomy also remains an option, although it carries the risk of positive surgical margins, potentially necessitating subsequent radical splenectomy (35). Splenic metastases present a particular challenge for radiotherapy due to their subdiaphragmatic location and respiratory motion, hence its limited clinical adoption. Reports describe the placement of three fiducial markers (FMs) via a transradial intravascular approach around the lesion to guide radiotherapy (36). Nevertheless, this technique is likely reserved as an alternative option for patients unsuitable for surgery or ablation.

Due to the rarity of isolated splenic metastasis in colorectal cancer, we report this case to contribute to the existing data and offer insights for future treatment. Splenic metastasis in colorectal cancer typically manifests as a terminal-stage disease. Its management centers on a multidisciplinary approach (MDT-guided therapy), integrating systemic treatment with potentially curative-intent local interventions.

For patients presenting with solitary splenic metastasis, curative therapies (radical surgery or ablation therapy) are associated with significant survival benefits.

Looking ahead, we propose the establishment of international multicenter collaborative registries to systematically aggregate clinical treatment data and outcomes, thereby generating large-scale, high-quality evidence. Concurrently, leveraging artificial intelligence and radiomics holds promise for refining prognostic models and predicting therapeutic responses, ultimately contributing to personalized, optimized treatment strategies.

## Data Availability

The original contributions presented in the study are included in the article/supplementary material. Further inquiries can be directed to the corresponding author.
